# The Utility of Resolving Asthma Molecular Signatures Using Tissue-Specific Transcriptome Data

**DOI:** 10.1534/g3.120.401718

**Published:** 2020-09-08

**Authors:** Debajyoti Ghosh, Lili Ding, Jonathan A. Bernstein, Tesfaye B. Mersha

**Affiliations:** * Immunology and Allergy, Department of Internal Medicine, University of Cincinnati, OH; † Division of Biostatistics and Epidemiology, Cincinnati Children’s Hospital Medical Center, Department of Pediatrics, University of Cincinnati, OH; ‡ Division of Asthma Research, Cincinnati Children’s Hospital Medical Center, Department of Pediatrics, University of Cincinnati, OH

**Keywords:** Asthma transcriptome, tissue-specific analysis, machine learning, pathways/networks, GWAS Catalog, Connectivity Map, ilincs

## Abstract

An integrative analysis focused on multi-tissue transcriptomics has not been done for asthma. Tissue-specific DEGs remain undetected in many multi-tissue analyses, which influences identification of disease-relevant pathways and potential drug candidates. Transcriptome data from 609 cases and 196 controls, generated using airway epithelium, bronchial, nasal, airway macrophages, distal lung fibroblasts, proximal lung fibroblasts, CD4+ lymphocytes, CD8+ lymphocytes from whole blood and induced sputum samples, were retrieved from Gene Expression Omnibus (GEO). Differentially regulated asthma-relevant genes identified from each sample type were used to identify (a) tissue-specific and tissue–shared asthma pathways, (b) their connection to GWAS-identified disease genes to identify candidate tissue for functional studies, (c) to select surrogate sample for invasive tissues, and finally (d) to identify potential drug candidates *via* connectivity map analysis. We found that inter-tissue similarity in gene expression was more pronounced at pathway/functional level than at gene level with highest similarity between bronchial epithelial cells and lung fibroblasts, and lowest between airway epithelium and whole blood samples. Although public-domain gene expression data are limited by inadequately annotated per-sample demographic and clinical information which limited the analysis, our tissue-resolved analysis clearly demonstrated relative importance of unique and shared asthma pathways, At the pathway level, IL-1b signaling and ERK signaling were significant in many tissue types, while Insulin-like growth factor and TGF-beta signaling were relevant in only airway epithelial tissue. IL-12 (in macrophages) and Immunoglobulin signaling (in lymphocytes) and chemokines (in nasal epithelium) were the highest expressed pathways. Overall, the IL-1 signaling genes (inflammatory) were relevant in the airway compartment, while pro-Th2 genes including IL-13 and STAT6 were more relevant in fibroblasts, lymphocytes, macrophages and bronchial biopsies. These genes were also associated with asthma in the GWAS catalog. Support Vector Machine showed that DEGs based on macrophages and epithelial cells have the highest and lowest discriminatory accuracy, respectively. Drug (entinostat, BMS-345541) and genetic perturbagens (KLF6, BCL10, INFB1 and BAMBI) negatively connected to disease at multi-tissue level could potentially repurposed for treating asthma. Collectively, our study indicates that the DEGs, perturbagens and disease are connected differentially depending on tissue/cell types. While most of the existing literature describes asthma transcriptome data from individual sample types, the present work demonstrates the utility of multi-tissue transcriptome data. Future studies should focus on collecting transcriptomic data from multiple tissues, age and race groups, genetic background, disease subtypes and on the availability of better-annotated data in the public domain.

While asthma is primarily recognized as a disease involving the lungs it has been increasingly understood to represent a systemic disease consisting of networks between various tissue/organs and associated with nasal, sinus, skin or allergic gastro-intestinal diseases which all exhibit inflammatory changes involving a broad spectrum of structural cells, adaptive and innate immune cells and circulating or tissue effector cells (Bjermer 2007). Crosstalk between upper and lower respiratory airways and other tissues, through inflammatory mediators, leads to systemic propagation of inflammation in multiple tissues resulting in disease progression. Genes differentially regulated in disease *vs.* control tissues can be used to explore the connectivity between disease, gene expression and perturbagens (candidate therapeutic agents) (Lamb *et al.* 2006). Previous gene expression studies have utilized biological samples from bronchial and alveolar macrophages, upper and lower airway epithelial cells and peripheral whole blood or other circulating blood cells to identify genes differentially expressed in asthma compared to healthy controls (Tsitsiou *et al.* 2012; Kicic *et al.* 2010; Choy *et al.* 2011). Although these studies produced lists of differentially expressed genes (DEGs), there tends to be little or no overlap among the lists of genes obtained using different tissues/cells suggesting disease-associated genes are likely to show tissue-specific expression patterns (Lage *et al.* 2008; Reverter *et al.* 2008). Most gene expression studies so far reported tissue combined approaches. Such an approach largely ignored the inherent gene expression heterogeneity of tissues/cells, and highly expressed genes rather than biologically relevant genes (but are too small to be detected in tissue-combined analysis) may be lost and potentially remain under-defined. Therefore, a tissue/cell-based gene expression analysis is necessary to track real driver genes and reduce potential confounders produced due to highly expressed genes from tissue/cell types. Thus, the analyses of genome-wide gene expression data originating from multiple tissue types would be helpful to understand tissue-specific or tissue-shared differentially regulated asthma genes. As the availability of genome-wide gene expression datasets has grown across major publicly data repositories, there is an increased interest to systematically mine these resources to identify novel patterns (Ramasamy *et al.* 2008).

In this study, we analyzed gene expression datasets available from GEO that were generated using multiple tissue/sample types (*e.g.*, blood, lymphocytes, upper and lower airway epithelial cells, lung biopsies, fibroblasts, macrophages and induced sputum) obtained from asthma patients and matched controls in order to obtain a tissue-resolved perspective of differentially regulated asthma-relevant genes and biologic pathways. This study has potential to (i) identify biomarkers from easily accessible non-invasive samples (*e.g.*, induced sputum) as surrogates for invasive, difficult to collect samples, (ii) connect GWAS-identified candidate genes to genome-wide gene expression results in asthma and (iii) identify potential therapeutic compounds for asthma based on tissue-specific and tissue-shared gene expression. In the current study we have used DEGs identified from different tissue/ sample types to select candidate perturbagens via Connectivity Map (CMAap), which currently covers > 1309 compounds connected with 7000 expression profiles.(Wang *et al.* 2015) This approach can identify drugs that affect the expression of common genes and identify candidate that could be potentially repurposed for systemic management of asthma (Ravindranath *et al.* 2015). However, how DEGs identified from different tissue/ sample types can potentially modify the list of perturbagens identified from CMAP analysis remains to be elucidated. Since DEGs vary across asthma-relevant tissue types, a better understanding of tissue-specific/ -shared DEGs underlying asthma may lead to the development novel drug candidates for to treat asthma.

## Materials and Methods

### Gene expression datasets description

**Figure 1:**
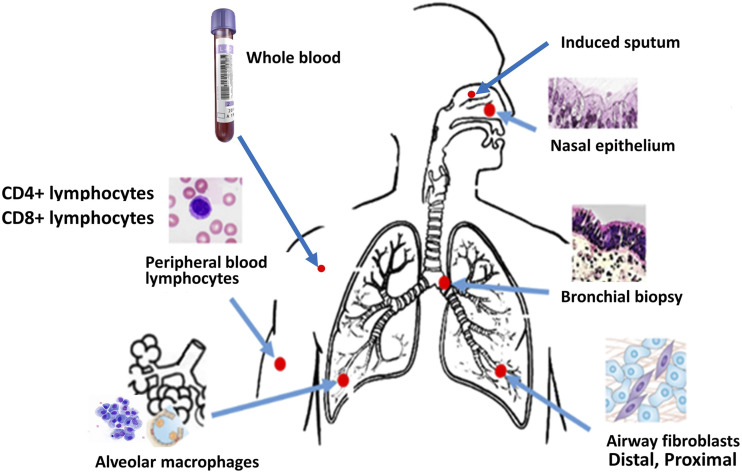
Asthma transcriptome data sources. Case and control gene expression datasets obtained from nasal epithelial cells, airway epithelial cells, bronchial biopsies, peripheral blood, CD4^+^ and CD8^+^ lymphocytes, airway macrophages, proximal and distal fibroblasts and induced sputum were used for the current study.

Data used in this study were retrieved from the publicly accessible Gene Expression Omnibus (GEO) database at the NCBI (http://www.ncbi.nlm.nih.gov/geo/) (Barrett *et al.* 2013; Edgar *et al.* 2002). We used the query terms “human [organism] AND asthma AND 2000/01:2019/06[Publication Date] to retrieve datasets from transcriptome studies comparing samples from multiple tissues (Figure 1) asthma patients with those of healthy individuals. Each individual dataset underwent consistent handling similar to when it was uploaded to GEO by the original study groups (preserving original normalization protocols, to maintain consistency with published reports).The following information was extracted from each study: (1) GEO accession, (2) sample type, (3) platform, (4) numbers of asthmatic and control individuals (Table 1). In total, 568,930 probes covering 25,000 genes were extracted for the analysis. The following asthma transcriptome data from the sample types were considered:

**Table 1:** Summary of transcriptomics datasets used for the present study. NCBI Gene Expression Omnibus (GEO) accession number, tissue/cell sample type, platform, sample size (excluding smokers and subjects using inhaled corticosteroids) have been shown for each study

	Sample type	GEO ID	Tissues/cell types	Platform	Case	Control
**I**	Airway Epithelium	GSE4302	Airway epithelial cells	Affy HG-U133	10	10
**II**	Airway Epithelium	GSE18965	Airway epithelial cells	Affy HG-U13A	9	7
**III**	Bronchial Biopsy	GSE15823	Endobronchial biopsies	HG_U95	4	4
**IV**	Bronchial Biopsy	GSE41649	Endobronchial biopsies	HG-U133	4	4
**V**	Macrophages	GSE22528	Alveolar macrophages	Affy HG-U133	4	4
**VI**	Macrophages	GSE2125	Alveolar macrophages	Affy HG-U133	15	15
**VII**	Fibroblasts	GSE27335	Proximal airway fibroblasts	Agilent	8	4
**VIII**	Fibroblasts	GSE27335	Distal lung fibroblasts	Agilent	8	4
**IX**	Lymphocytes	GSE31773	CD4+ T-cells	Affy HG-U133	12	8
**X**	Lymphocytes	GSE31773	CD8+ T-cells	Affy HG-U133	12	8
**XI**	Nasal Epithelial	GSE44037	Nasal epithelial cells	Affy HG-U133	6	6
**XII**	Whole blood	GSE69683	Peripheral Blood	Affy HG U133	411	84
**XIII**	Induced sputum	GSE76262	Induced sputum (whole)	Affy HG U133	118	21

#### Nasal epithelial cells (NECs):

Nasal epithelial cells were obtained by nasal brushing. Six asthmatics and 6 healthy controls were used (GSE44037). NECs are of particular interest for studies in children due to their easy accessibility during clinical visits (Poole *et al.* 2014).

#### Airway epithelium:

Airway epithelial gene expression data were obtained from GSE4302 (asthmatics= 10, controls = 10) and GSE18965 (asthmatics= 9, controls = 7). Subjects using inhaled medication or tobacco smoke have been removed from our analysis.

#### Bronchial biopsy:

Data were obtained from asthma (n = 4) and control (n = 4) subjects (GSE15823) and asthma (n = 4) and control (n = 4) subjects (GSE41649). Data related to treatment outcome was not included. The samples typically contained mucosal and epithelial/sub-epithelial tissue (0.3 mm^2^ to 0.5 mm^2^) (Laprise *et al.* 2004; Choy *et al.* 2011; Chamberland *et al.* 2009; Labonté *et al.* 2008). Bronchial epithelium is a source of cytokines and chemokines which plays a key role in recruitment of inflammatory cells into tissues from the circulation.

#### Proximal airway fibroblasts:

We used asthmatic (n = 8) and control (n = 4) subjects (GSE27335) involving fibroblast samples obtained by bronchoscopy. Proximal airway fibroblasts are known to synthesize more collagen and eotaxin-1 than distal fibroblasts. Fibroblasts play key roles via an IL-13 induced mechanism involving TGF-β1 and MMPs leading to and lung remodeling in asthma (Ingram *et al.* 2011).

#### Distal airway fibroblasts:

Matched case-control distal lung fibroblast pairs were isolated from above-mentioned subjects’ distal lung from GSE27335 (asthmatics= 8, controls = 4). Distal lung fibroblasts exhibit a distinct phenotype, proliferate faster and express higher levels of α-smooth muscle actin compared to proximal airway fibroblasts. Distal airways demonstrate distinctly different fibrotic property compared to the proximal segments. Excessive deposition of extracellular matrix is particularly prominent in the proximal parts compared to proximal airways.

#### Alveolar macrophages (AMs):

Two GEO datasets GSE22528 and GSE2125 have been used for the present study which allowed us to utilize transcriptome data containing 10 cases and 223 controls, and 30 cases and 547 controls respectively. To generate transcriptome data, AM cells from broncho-alveolar lavage fluid of asthmatics and control subjects were used to perform homogeneity checks by flow cytometry prior to RNA isolation and microarray experiments performed by the investigators. Alveolar macrophages constitute a unique subset of pulmonary macrophages, which serve as a first line of defense against foreign invaders to the lung tissue.

#### Blood and lymphocytes:

Dataset GSE69683, which includes asthmatics (n = 399; severe and non-severe) and non-asthmatics (n = 101) excluding smokers, were used for this analysis. GSE 31773 was also used to identify asthma associated DEGs from CD4+ and CD8+ lymphocytes. This dataset was generated using CD4+ and CD8+ T cells isolated from peripheral blood of asthmatics (N = 18) and controls (N = 8) by negative selection and magnetic cell separation procedures followed by purity assessments using flow cytometry. Blood contains plasma and corpuscles, each paying multiple roles in inflammation. Peripheral blood mononuclear cells (PBMC) consist of lymphocytes (T cells, B cells, NK cells) and monocytes. TH2 cells represent a phenotype of T lymphocytes that upon activation generate IL-4, IL-5 and IL-13 cytokines in both peripheral blood and bronchial mucosa of asthma patients, leading to local tissue eosinophilia, generation of specific IgE antibody responses and airway hyperresponsiveness/ inflammation.

#### Induced sputum:

We obtained transcriptomic data from GSE76262 which was generated using sputum cells collected from asthmatics (n = 118) and matched healthy controls (n = 21). Induced sputum is an easily accessible non-invasive approach for obtaining cellular (epithelial cells, eosinophils, lymphocytes, neutrophils and macrophages) and non-cellular (cytokines, chemokines and other secreted products/ inflammatory mediators) components from the lower respiratory tract.

### Analysis workflow

**Figure 2:**
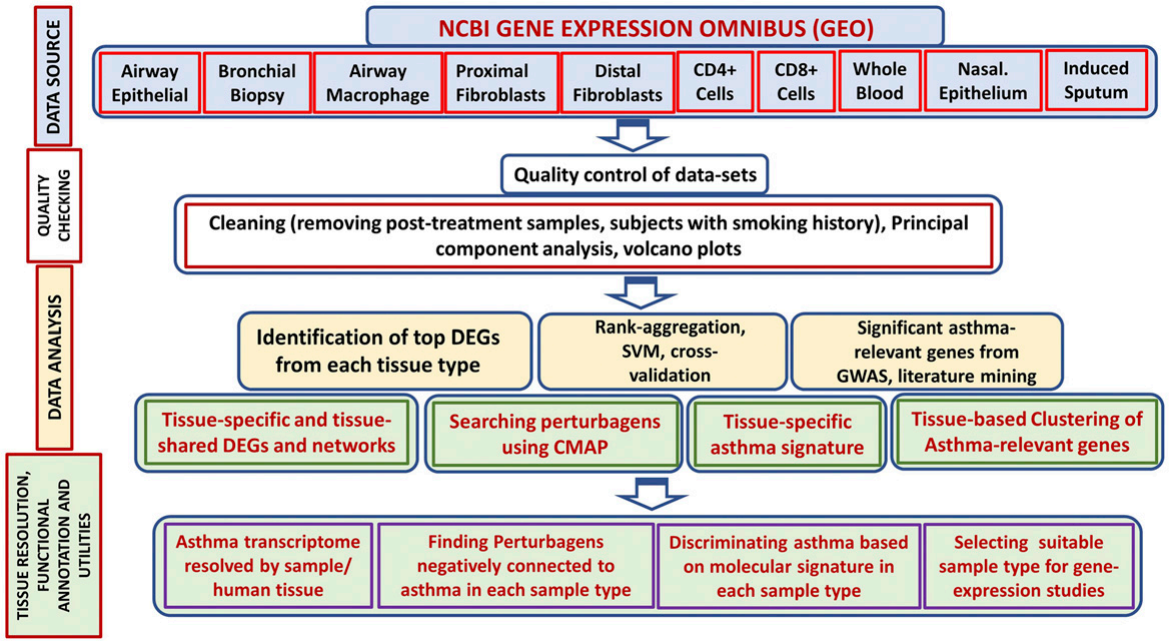
Major steps to identify and analyze tissue specific asthma gene expression data. Individual datasets were obtained from GEO. Differentially expressed asthma-relevant genes identified from each tissue types were used to find tissue/ cell-specific genes and networks, and discriminatory gene-sets to classify samples into normal *vs.* asthma classes and to predict asthma disease state. In addition, differentially expressed genes identified from each sample/ tissue type were linked with GWAS catalog data and Connectivity Map resources to identify novel drug candidates.

The workflow for data analysis *is* illustrated in Figure 2. Genome-wide transcription data obtained from the seven different tissues/cell sample types include airway epithelial, bronchial biopsy, nasal epithelial, alveolar macrophages, distal lung fibroblasts, proximal lung fibroblasts, CD4+ lymphocytes, CD8+ lymphocytes, whole blood and induced sputum. Tissue-specific and tissue-combined analyses were performed to identify differentially expressed genes.

The transcriptome datasets underwent careful quality control. If a single GEO dataset contained data from multiple tissues/ sample types (*e.g.*, GSE31773 contains CD4+ and CD8+ cells and GSE27335 contains proximate and distal macrophages), such datasets were further divided based on tissue/sample types. This generated 13 datasets, each with cases and controls, for downstream analysis (Table1). Finally, data from subjects on asthma medication that could potentially change the gene expression were also removed.

### Probe level analysis

Each dataset was downloaded from GEO for further tissue-specific analysis. Differentially expressed genes were identified for each individual dataset at the probe level using limma R packages from the Bioconductor project (https://www.bioconductor.org/). Genes in each dataset were arranged by statistical significance. Among the top DEGs based on p-value, those showing a fold-change of 1.5 (up- or down-regulated) were considered for further analysis.

### Gene-level overlap between tissue/cell types

The probe IDs are mapped to NCBI generated annotations, which is available from the UCSC genome browser site. Annotations such as gene symbol and gene title are derived by extracting stable sequence identification information from the platform and periodically querying against the Entrez Gene and UniGene databases to generate consistent and up-to-date annotation. Then, the transcripts showing transcript IDs that did not have a corresponding gene symbol were exported to DAVID (http://david.abcc.ncifcrf.gov/home.jsp) for gene ID conversion (Huang *et al.* 2009a, 2009b).

### Support vector machine (SVM) model

SVM is one of the most popular supervised learning methods for analyzing data and recognizing patterns.(Fekete *et al.* 2012) The objective of the support vector machine algorithm is to find a hyperplane that classifies data points (potentially case *vs.* control). Given labeled training data (supervised learning), the algorithm generates an optimal hyperplane that can be used to categorize new datapoints. SVM therefore represents a powerful technique for general (nonlinear) classification, regression and outlier detection and has been widely used in bioinformatic applications such as finding signature transcripts. The SVM function in R package e1071 was used to build the statistical prediction model with C-classification and radial kernel, where the parameters were tuned to give the best prediction results.(Lepre *et al.* 2013)

Since individual tissue/ cell type can be associated with multiple gene expression datasets from different studies that used different microarray platforms, probe level information was mapped to gene level in order to aggregate differentially expressed asthma genes within tissue/cell types. This procedure was done for each dataset. DEGs at gene level were taken as the probe with the smallest p-value. The ‘RobustRankAggreg’ package (a powerful rank-based data aggregation tool capable of combining ranked lists coming from different sources and platforms such as different microarray chips) of the R software was then used to combine and establish a consensus rank of DEGs per tissue at the gene level (Pihur and Datta 2009). SVM was used to ascertain whether top molecular profiling ‘signature’ genes can be used to discriminate asthma patients from controls in various tissue types. For each dataset, we randomly select about 4/5 of the sample as the training dataset and the rest as the testing dataset. We used the top ranked DEGs in each training dataset to build an SVM model with 10-fold cross-validation and test it in the testing dataset. The prediction accuracy and kappa coefficient were reported in both the training and testing datasets.

### Pathway and regulatory network analysis

Differentially expressed genes by tissue/cell types between asthma patients and normal controls were further investigated using Ingenuity Pathway Analysis (IPA) software (Qiagen, USA). The IPA methodology compares proportional representation of genes from a defined test set in a canonical pathway (a known, well-characterized pathway), compared to the proportional representation of the pathway genes in the entire set of known genes. The p-value is calculated using a right-tailed Fisher Exact test and indicates the likelihood of the pathway association under the random model. Pathway/networks with a score >2 have >99% confidence that the genes included in the network are not generated by chance. The score represents a numerical value to rank networks according to their level of relevance to the ‘Network Eligible’ molecules in the dataset considering the number of ‘Network-Eligible’ molecules within the network and its size, as well as the total number of ‘Network-Eligible’ molecules analyzed and the total number of molecules in the Ingenuity Knowledge Base that could potentially be included in networks. Finally, the networks are ordered according to their score, with the highest scoring network displayed at the top. The network Score is based on the hypergeometric distribution and is calculated with the right-tailed Fisher’s Exact Test.

### Linking DEGs with asthma genes identified from GWA Studies

There were 25 asthma studies resulting 38 genomic regions from GWAS Catalog (accessed July 2019).(Hindorff *et al.* 2009). Inclusion of asthma GWAS catalog-based associations was limited to those studies with P values of less than 5x10^−8^ (http://www.ebi.ac.uk/gwas/). Asthma-relevant genes were also identified from published literature using literaturelab (Accumenta, USA)(Febbo *et al.* 2007). Gene level expression of asthma-relevant genes was averaged over cases or controls in each dataset and correlation coefficients were calculated across datasets, which was used to run hierarchical clustering to identify groups of tissue/cell types that cluster more closely than others.

### Connectivity Map (CMap) analysis using tissue-specific DEGS

Next, we used Connectivity Map (CMap) analysis approach to explore potential drugs targeting asthma by systematically mining functional connections between asthma disease, DEGs, and perturbagens (Lamb *et al.* 2006). Differentially expressed genes were divided into two parts, one for upregulation and the other for downregulation. The CMap analysis was performed through the web interface CLUE (https://clue.io/), a cloud-based platform was used to analyze perturbation-driven gene expression signatures, following a standard protocol described by Wang *et al.*(Wang *et al.* 2019; Subramanian *et al.* 2017). Briefly, the “Query” and “Touchstone” applications of CLUE were used to identify reciprocal connectivity (negative correlation) between asthma and perturbagens where up- and down-regulated genes were used inversely as inputs to identify negative correlation (Wang *et al.* 2019) (Lex *et al.* 2014). CMap instance was measured by an enrichment score, which ranged from −1 to 1, and a permutation P-value. For the current analysis, any connectivity score of below -0.85 or above 0.85 was considered for this analysis (Subramanian *et al.* 2017).

### Data availability

The data supporting this work is publicly available from NCBI GEO (Gene Expression Omnibus): https://www.ncbi.nlm.nih.gov/gds/?term=asthma. Supplemental material available at figshare: https://doi.org/10.25387/g3.11935959.

## Results

### Tissue-combined and tissue-resolved approaches

Tissue/ cellular samples used in this study included airway epithelial cells, bronchial biopsy, alveolar macrophages, peripheral blood, purified peripheral blood CD4+ lymphocytes, CD8+ lymphocytes, proximal lung fibroblasts, distal lung fibroblasts, induced sputum and nasal epithelium samples (Table 1). In an initial tissue-aggregated assessment (tissue-combined approach), top significant transcripts showed an inability to discriminate between normal and asthma subjects (Supplementary Figure S1A) by Principal Component Analysis (PCA) indicating the importance of tissue- specific analysis in asthma. Tissue-resolved analysis approach was therefore followed subsequently. Tissue-specific differentially expressed signature genes (number ranged from 126 to 1060) were then obtained for each of the seven tissues. Significant genes showing absolute fold change values of 1.5 or higher were considered for further analysis.

**Figure 3:**
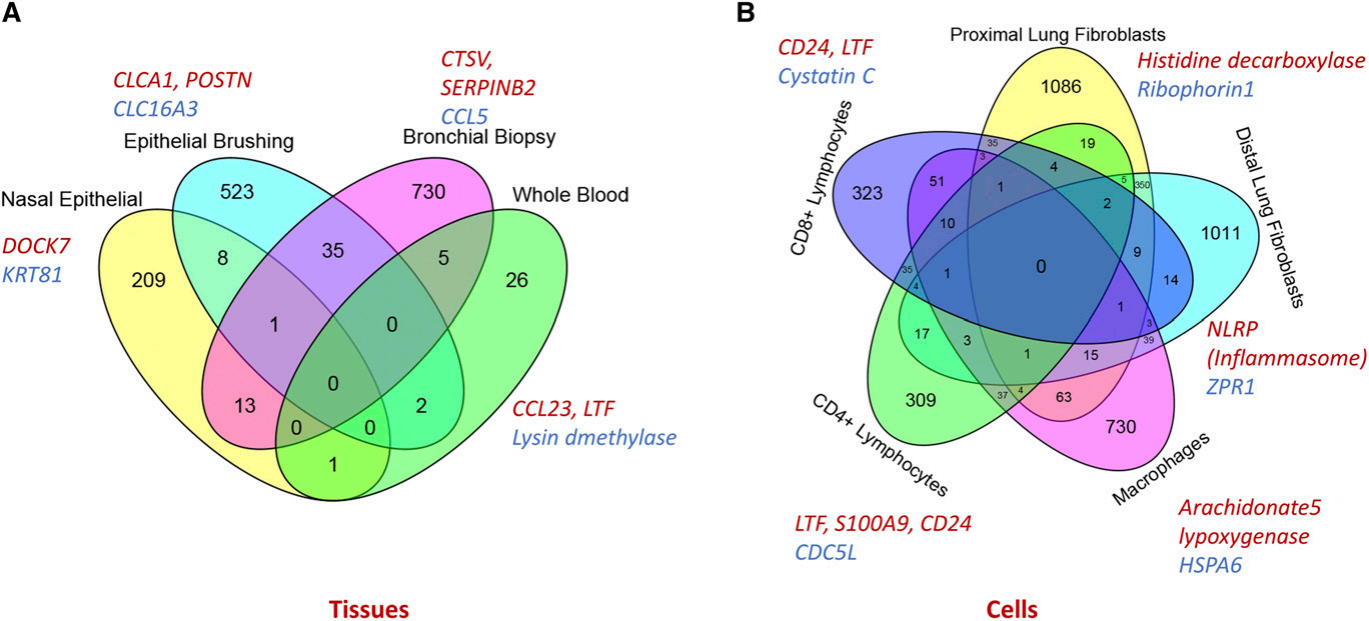
Gene-level overlap: Venn diagram showing overlap of differentially regulated genes identified from tissue samples (A; airway epithelial, bronchial, nasal and whole blood samples) and isolated cellular samples (B; lymphocytes, fibroblasts and macrophages). There is considerable overlap at the gene-level between airway epithelial and bronchial biopsy samples. However, each sample type shows a unique asthma-relevant gene expression pattern; DEG sharing is not observed between all samples. Top significant tissue/ cell-specific DEGs (red up-regulated, blue down-regulated) have been shown for each sample type.

When the data were analyzed tissue by tissue (tissue-resolved approach), a clear distinction between normal and asthma subjects was observed. The lowest differentially expressed gene count was observed in bronchial biopsy tissues (173 DEGs) while the highest was observed in the nasal biopsy tissue (1320 DEGs). PCA showed clear separation of asthmatics from controls in tissue specific analysis (Supplementary Figure S1 B-D). Jaccard similarity index was used to determine similarity between various tissues, nasal and airway epithelial cells show the highest (0.61) while CD4+ lymphocytes and Bronchial biopsies (0.01) showed the lowest Jaccard similarity index, respectively. Differentially expressed genes were identified from datasets representing multiple sample types consisting of tissues (*e.g.*, airway epithelial, bronchial, nasal) and isolated cells (*e.g.*, CD4+ lymphocytes, CD8+ lymphocytes, macrophages, isolated fibroblasts). Figure 3A and 3B demonstrate the overlap of DEGs identified from tissue and cellular samples, respectively. Differentially expressed genes identified from different tissue types were compared to obtain tissue-specific and tissue-shared genes and pathways relevant for asthma as described below:

#### Airway epithelial samples:

In airway epithelial tissue, increased expression of the chloride channel calcium-activated, family member 1 (*CLCA1*) (6.2 fold), periostin (4.4 fold), and serine peptidase inhibitor clade B member 2 (*SerpinB2*) (also known as plasminogen activator inhibitor-2) (3.5 fold) was observed. Originally described as a calcium-activated chloride channel, CLCA1 likely functions indirectly in chloride transport and has previously been reported to be up-regulated in asthma. Periostin is an integrin ligand and extracellular matrix protein with roles in cell adhesion, cell motility, and matrix remodeling. SerpinB2 is a member of the serpin class of proteases and functions to inhibit plasminogen activation and to promote fibrin formation and deposition. Among the other most differentially expressed genes in lung airway epithelial brushings were three mast cell markers: carboxypeptidase A3 (3.4 fold induced), tryptase β2 (2.2 fold induced), and tryptase α/β1 (2.1 fold induced).

The transforming growth factor beta (TGF-β) signaling pathway seems to play a major role in the manifestation of asthma, most notably in the airway remodeling process. TGF-β stimulates fibroblasts to synthesize and secrete proteins of the extracellular matrix in patients with severe asthma may induce smooth-muscle hypertrophy (Ohno *et al.* 1996; Cohen *et al.* 2000). In addition to the TGF-β pathway, extracellular signal regulated kinase (ERK) plays a major role in airway smooth muscle activity by virtue of its upstream activation of cyclin D1 promoter activity which is required for DNA synthesis in airway smooth muscle cells (Ramakrishnan *et al.* 1998; Zhou and Hershenson 2003). Consistent with this observation that ERK is critical for signaling of airway smooth muscle cell activity in asthma, bronchoalveolar lavage fluid from asthma patients has been shown to augment ERK activation, increase cyclin D1 protein abundance, DNA synthesis, and proliferation of cultured human airway smooth muscle cells (Naureckas *et al.* 1999). One very significant finding is the upregulation of the estrogen pathway in the airway epithelium of asthma patients. Estrogen receptors are found on numerous immunoregulatory cells and estrogen’s actions skew immune responses toward allergy. The role of estrogen receptor in asthma is being increasingly recognized. For example, the G-protein-coupled estrogen receptor agonist has been found to suppress airway inflammation through IL-10 in a mouse model of asthma.

#### Lymphocytes:

DEGs unique to CD4+ and CD8+ cells as well as common DEGs were analyzed. The top up-regulated genes were related to bacterial resistance, solute transport and calcium signaling. While asthma involved pathways like NFAT in regulation of the immune response and other lymphocyte activation pathways including signaling through IL-4, IL-8, IL-3 and IL-9 in CD8+ cells, the most prominent pathway in CD4+ cells was VDR/RXR activation signaling. Activation of ERK pathway was also noted to be the top common pathway in lymphocytes, followed by Immunoglobulin synthesis and inflammatory cytokine synthesis pathways.

#### Macrophages:

The two datasets generated using bronchial macrophage cells, showed that ERK was the main regulatory network. In an individual study, Madore *et al.* identified differentially expressed asthma genes related to stress and immune responses using airway macrophage cells obtained from asthma and healthy controls (Madore *et al.* 2010).

#### Fibroblasts:

The fibroblast transcripts focused on the differences in gene expression between distal and proximal fibroblasts. Distal Lung Fibroblasts exhibited a higher basal activation of *SMAD3* and MAPK8 compared to its proximal counterpart.

#### Nasal epithelial cells:

Nasal epithelial cell samples revealed CCL chemokines as the most biologically relevant DEG candidates exhibiting higher expression in asthma patients and were associated with increased eosinophil and monocyte chemotaxis. In addition to prominent eosinophilic inflammation, increased *IL1RL1* expression was also found.

#### Tissue-shared DEGs, the multi-tissue factors:

The *ATXN2* gene was significantly upregulated in airway epithelial brushing, bronchial biopsies and proximal as well as in distal lung fibroblasts. Interestingly, a variant of this gene was recently found to be associated with compromised lung function (Wain *et al.* 2017; Soler Artigas *et al.* 2015). In addition, upregulation of the integrin ITGB1 at the mRNA level has been demonstrated in airway smooth muscle cells in the asthma model group (Álvarez-Santos *et al.* 2016). The integrins are important cell surface adhesion receptors that recognize extracellular matrix components known to be altered in airway cells in asthma. Intrinsic differences specifically related to proliferation, differentiation, and migration contribute to the dysregulated bronchial epithelial cell response to injury.

### Functional enrichment, pathway-level overlap and networks

**Figure 4:**
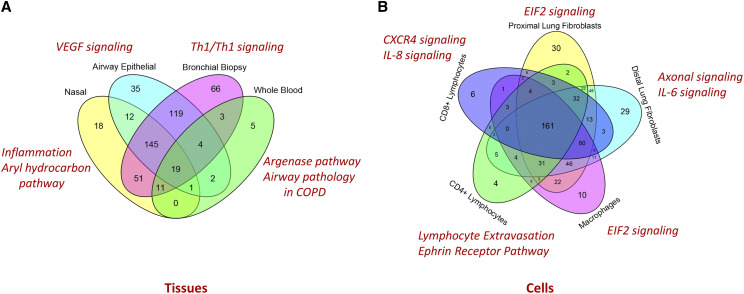
Pathway-level overlap in tissue (A) and isolated cellular level (B) samples: Argenase pathway, Th1/Th2 signaling, VEGF signaling and Inflammation and Aryl hydrocarbon pathways were most predominant on blood, biopsy, airway epithelial and nasal samples respectively. EIF2 signaling was relevant for macrophages and proximal fibroblasts, while lymphocyte extravasation (adhesion and diapedesis), IL6 pathway and IL-8 signaling pathways were very relevant for CD4+, distal lung fibroblasts and CD8+ cells respectively. In addition, Glucocorticoid Receptor Signaling, Clathrin-mediated Endocytosis Signaling were top significantly enriched pathways in all tissue types. IL-1beta and ERK signaling pathways were common across a wide range of tissue types. Chemokine signaling (in nasal epithelium) were the most significant pathways.

In order to gain further insight into the functional significance of tissue-specific and/or shared DEGs, a gene set enrichment analysis based on gene ontology annotations was performed. Immune system process and cell adhesion were the GO terms that were over-represented (8 genes each) within our list of DEGs. Interestingly, out of the 8 genes representing an immune system process, only 4 (*ALOXA15,* *ITGB2, CD44 and EDNRA*) had previously been associated with asthma. The differentially expressed genes between asthma and controls in each tissue type were used to identify gene networks by the IPA software application. Two gene networks were identified with a network score of >2. The highest scoring network had a network score of 21 and was associated with the network functions of cellular movement, immune cell trafficking and inflammatory response. Figure 4A and 4B demonstrate the overlap of enriched pathways in tissue and isolated cellular samples in asthma.

Pathway analysis identified autophagy, VEGF (vascular endothelial growth factor) Signaling, Paxillin Signaling, Actin Cytoskeleton Signaling and ERK5 Signaling as the top canonical signaling pathways in lower airway epithelial samples. Interestingly, VEGF Signaling, has been designated as a very important pathway in asthma by inducing remodeling and enhancing TH2-mediated sensitization and inflammation in the lung (Lee *et al.* 2004). Paxillin and actin cytoskeletal signaling have been found to be important for bronchial tissue contraction and airway remodeling in asthma. However, thus far, the role of the Pax gene (encoding paxillin protein) in asthma has not been sufficiently explored. Analysis of the lymphocyte data using IPA pathway analysis tool indicated that activation of multiple pathways related to T-cell activation, inflammatory cytokine and immunoglobulin synthesis were important. In lymphocytes, IgE synthesis, cytokine signaling, and ERK-mediated cell proliferation have been found to be the major driving factors for asthma. For macrophages, pathway and network analysis identified IL-12 and ERK as the major hub genes or nodal genes indicating the role of sustained inflammation via the IL12 pathway and control of cell proliferation via the ERK pathway. For fibroblast samples, the up-regulation of extracellular matrix-associated molecules, actin binding and cytoskeletal protein molecules has been previously reported in proximal and distal lung fibroblasts respectively. Genome-wide gene expression differences between these two populations of regional lung fibroblasts might explain different responses to cell injury, cell regeneration, and subsequent airway remodeling in the lung. We noticed that top significant asthma-relevant networks are distinctly different between airway macrophages, proximal lung fibroblasts and distal lung fibroblasts of asthmatics indicating that although all of these samples were obtained from airway compartment they demonstrate different asthma-relevant genes and networks (Supplementary Figure S2).

Interestingly, although epithelial gene expression patterns differ between nasal and bronchial samples, they exhibit significant similarities at the functional level. For example, the genes *CTSC,* *ELAVL2, IL13RA2, IL1R2, IRX4* had higher expression in nasal compared to bronchial epithelium, but both demonstrated activated functional clusters important for cell-mediated immune responses, hematological system development and function as well as immune cell trafficking.

We also compared the pathway-level overlap between samples to assess whether easily accessible sample types (such as nasal epithelium and induced sputum) could be used as surrogates for more invasive sample types (such as airway epithelial brushing and bronchial biopsy sample). Airway epithelium can be partially represented by the nasal samples, rather than from induced sputum which might partially represent bronchial samples at the pathway-level indicating the significance of easily accessible samples as surrogates for less accessible clinical samples (Supplementary Figure S3). Therefore, the nasal epithelial cells are easier to obtain and may act as a surrogate for airway epithelium (calculated Jaccard coefficient = 0.43) in asthma studies as previously indicated (McDougall *et al.* 2008).

### Linking tissue-specific DEGs with asthma GWA studies

**Figure 5:**
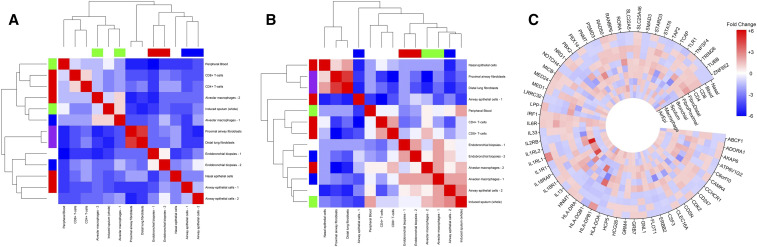
Tissue-based expression of asthma-relevant genes identified by (A) GWAS retrieved from GWAS-catalog (European Bioinformatics Institute) and by (B) literature mining identified by using Literature Lab (Accumenta, USA). The results showed that asthma-relevant genes can be clustered by their expression in target cells/ tissues. Macrophage samples are closely associated with induced sputum samples, whereas CD4+ and CD8+ lymphocyte samples cluster with peripheral blood samples in both (A) and (B) samples. Bronchial biopsy samples cluster together. The tissue-based clustering of asthma-relevant genes has been very clearly demonstrated in case of genes identified by literature-mining (B). A gene-level circular heatmap demonstrating tissue-wise expression of GWAS-identified genes have been shown in panel C.

To further assess the inter-tissue expression differences of asthma-relevant genes we analyzed the expression of asthma-associated genes identified from GWAS and from published asthma literature. GWA studies have identified many genetic variants associated with complex diseases like asthma. However, gene regulatory architectures differ between tissues, and the enrichments for regulatory variants among GWAS SNPs are more pronounced in disease-relevant tissues (Roadmap Epigenomics Consortium *et al.* 2015). Thus the relevance of significant GWAS loci in altering biological processes (such as up/down regulated genes, biological process, functions and networks) that result in risk for or protection from the disease remains to be a key question, which requires access to the disease tissues of interest (Koester and Insel 2016). Immunoglobulin genes and IL-1 signaling pathways, which are key regulatory pathways in asthma, were identified by GWAS and tissue-based gene expression analysis (Torgerson *et al*., 2011). Overlaping DEGs between our study and those asthma genes identified from GWA studies clustered by their expression in asthma-relevant cells/ tissues (Supplementary Figure 5A and 5B). A gene-level circular heatmap shows tissue-wise DEGS and GWAS-identified genes (Figure 5C).

### Predicting asthma risk using easily accessible surrogate clinical samples

Next, we attempted to ascertain whether more accessible clinical sample types (such as blood, induced sputum, airway macrophage) could be used to discriminate asthma from controls thereby potentially useful for predicting asthma. Prediction analysis using SVM was used to determine a minimal set of genes that could best discriminate asthmatics from controls. Representative asthma signature for peripheral blood, Airway Macrophage and Induced sputum that could discriminate asthma from control with > 80% predictive accuracy have been shown in Table 2. Interestingly, in our SVM model, the expression of only a set of 10 genes could discriminate cases from controls with 96% predictive accuracy.

**Table 2:** SVM-generated classifiers (Asthma Gene Expression signatures) for sample types (blood, Macrophage, induced sputum) that are widely used to study the pathology of asthma. Percent predictive accuracy for each classifier has also been indicated

Tissue Type	Asthma Gene Signature (for >80% predictive accuracy)	% correct prediction
**Blood (GSE69683)**	MYD88, MYL12A, BOLA2, HBXIP, ACSL1, VPS24, GPM6B, F5, PDE7A, AB11FIP3, ZNF785, TCF4, RAB11A, GPR109B, GNAQ, DIS3L2,, SLC7A5P1/P2, TSR2, AMICA1, CR1, QRICH1, SERBP1, ZNF37A, DERL2, ZBTB20, CDC42SE1, NSMAF, KIAA0562, NT5C2, DAPP1, ATP6V0E1, LOC203274, TMEM59, NAPEPLD, ANXA3, WIPI2, ATAD5, LARS, PTBP1, SPG7, DNAJC16, IL6ST, CLN8, SAT1, EVI2B, MRPS5, AQP9, RPP14, ZNF747, GAPT	83
**Macrophage Cells (GSE2125)**	KBTBD2, UPF3A, MLLT10, PSPC1, ST3GAL1, ARFRP1, TRIM14, EIF4G1, BM25, CDK11A/B,	83
**Induced Sputum (GSE76262)**	IL18R1, TLR7, GPR85, LIMA1, SPINT1, EIF1AY, PDCD6, MGAT4A, CSTA, LOC100129845	96

### Utilizing tissue-specific DEGs to identify candidate ASTHMA therapeutics

Asthma has been recognized as a systemic disease consisting of networks between various tissues showing inflammatory changes involving a broad spectrum of structural cells, adaptive and innate immune cells. Therefore, the DEGs identified from each tissue were used to “connect” asthma with candidate therapeutics using computational drug repurposing approach (Subramanian *et al.* 2017). For this, tissue-specific asthma-associated gene expression signatures were matched to previously characterized perturbagens that may potentially reverse the signature in multiple cell/ tissue sample types. Table 3 shows perturbagens associated with three or more asthma tissue/sample types, occurrence (number of connected asthma tissues), and their association with allergy/ asthma/ lung diseases (if known). Perturbagens can be ‘genetic’ (gene knock-down and over-expression; 20 candidates) or ‘pharmacologic compound’ (*e.g.*, entinostat, BMS-345541, calyculin, importazole and topotecan; 5 candidates), while pharmacologic compound could be sub-categorized into biologic or chemical drugs. The connectivity scores of perturbagens are presented in supplement S1.

**Table 3:** Perturbagens associated with at least three asthma-relevant asthma sample types. occurrence (connection to number of asthma tissues), associated asthma tissue/ sample type and their known association with allergy/ asthma/ lung diseases (known/ unknown) have been Mentioned

Perturbagen	Occurrences	Associated samples	Indicated in allergy/ asthma/ lung disease
KLF6 (Kruppel like factor 6)	5	AirEpi, Macrophages, FibroDistal, CD8+, Nasal	Yes
BCL10 (B-cell lymphoma/ leukemia 10)	4	Macrophages, FibroDistal, CD4+, Blood	Yes
HOXB13 Homeobox protein Hox-B13	4	Macrophages, Sputum, FibroDistal, Nasal	No
IFNB1(Interferon beta 1)	4	Sputum, FibroDistal, CD8+, Blood	Yes
Entinostat	4	Macrophages, FibroDistal, CD8, Nasal	No
ATOX1(Antioxidant 1 Copper Chaperone)	3	FibroDistal, CD4, FibroProximal	No
BAMBI(BMP and activin membrane bound inhibitor)	3	Macrophages, Sputum, Blood	Yes
BMS-345541	3	FibroDistal, CD8+, Nasal	Yes
CCNL1 (cyclin L1)	3	Macrophages, FibroDistal, CD8+	No
CDCA8 (cell division cycle associated 8)	3	AirEpi, CD8+, Blood	No
DHX8 (DEAH-box helicase 8)	3	Sputum, Bronchial, Blood	Yes
DTX2 (deltex E3 ubiquitin ligase)	3	AirEpi, Sputum, Nasal	No
KLF3 (Kruppel like factor 3)	3	Bronchial, FibroDistal, CD8+	Yes
LASP1 (LIM and SH3 protein 1)	3	AirEpi, Macrophages, CD8+	Yes
LOXL1 (lysyl oxidase like 1)	3	AirEpi, Sputum, Blood	Yes
PPP2R3C (protein phosphatase 2 regulatory subunit B’’gamma)	3	AirEpi, Macrophages, Blood	Yes
PREB(prolactin regulatory element binding)	3	Macrophages, FibroDistal, FibroProximal	No
PUF60 (poly-U binding splicing factor 60)	3	Macrophages, CD8+, Nasal	No
SORBS3 (sorbin and SH3 domain containing 3)	3	Macrophages, FibroProximal, Nasal	No
TRIP10 (Thyroid Hormone Receptor Interactor 10)	3	Macrophages, Bronchial, FibroDistal	No
XPO7 (Exportin 7)	3	AirEpi, Sputum, Bronchial	Yes
YWHAZ	3	AirEpi, Bronchial, Nasal	No
Calyculin	3	Macrophages, FibroDistal, Blood	No

**Figure 6:**
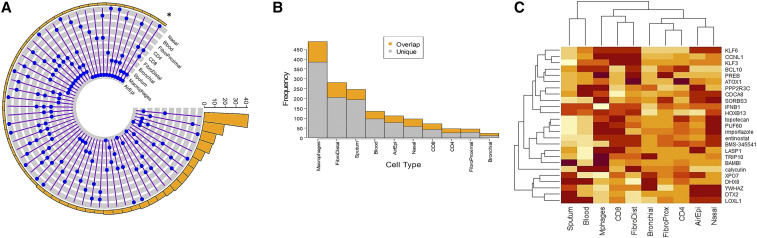
Asthma tissue transcriptome linked to drug repurposing. The blue connected dots (in A) indicate overlap between sample types (gray tracks) while the asterisk showing perturbagen connecting maximum tissue types. The bar chart (B) indicates the number of unique perturbagens for each sample type with overlapped portion in orange. Clustered heatmap (C) connecting asthma-relevant tissue/ samples to perturbagens has been generated using respective connectivity S1 Table scores) in each tissue. Asthma associated up and down-regulated genes identified form different tissue/ sample types were used to identify perturbagens that can potentially reverse asthma signature in respective sample type.

We found that no L1000-characterized perturbagen is shared by all asthma tissue sample types. The candidate perturbagen shared by most sample types is KLF6 (5 sample types), followed by BCL10, HOXB13, IFNB1 and Entinostat (4 sample types each). While 20 other perturbagens (ATOX1, BAMBI, BMS-345541, CCNL1, CDCA8, DHX8, DTX2, KLF3, LASP1, LOXL1, PPP2R3C, PREB, PUF60, SORBS3, TRIP10, XPO7, YWHAZ, calyculin, importazole, topotecan) are associated with three different sample types, large numbers of perturbagens are connected to either two (171 perturbagens) or only one (1126 perturbagens) sample type. Several top-ranking perturbagens have been indicated by previous studies to be involved in allergy/ asthma or other lung diseases (*i.e.*, Chronic Obstructive Pulmonary Disease; COPD). Figure 6 depicts unique and shared perturbagens connected to asthma status in different asthma sample types. The blue connected dots (6A) indicate shared candidates between sample types (gray tracks) with the asterisk showing perturbagen connecting maximum samples, while the bar chart (6B) indicates the number of unique perturbagens for each sample type. Finally, figure 6C represents a clustered heatmap generated using the connectivity scores (shown in supplementary table s1) of perturbagens (25 significant perturbagens listed in table-3) depicting the connections between disease, candidate perturbagens and tissue-based gene signatures of asthma. Connectivity score values of perturbagens were generated by touchstone application of CLUE (www.clue.io). Further details about their characteristics have been listed in Supplementary S2. Analysis of functional significance of the perturbagens showed their roles in immune function, cellular transport function, apoptosis and inflammation, with several agents showing one or more overlapping functions. The results clearly demonstrated that the connectivity scores of perturbagens can vary depending on input source tissue used to identify asthma-relevant DEGs. For example, the genetic perturbagen can be relevant for distal fibroblasts, CD+ lymphocyte and nasal epithelium, but may not be relevant for proximal fibroblasts and CD4+ lymphocytes. Similarly, Macrophages, the chemical perturbagen entimostat may be relevant for distal fibroblasts, CD8+ lymphocytes and nasal epithelium, but may actually have an opposing effect based on sputum and whole blood asthma signatures. Collectively our results indicate a critical role of tissue sample (used in generating gene expression data) in identifying connected drug candidates.

## Discussion

Tissue-specific gene expression plays a fundamental role in multi-cellular pathobiology (Song *et al.* 2013). However, until recently direct comparative analysis across multiple tissue types was not possible due to the limited number of gene expression data available from public domain. Herein, we studied tissue-specific DEGs in multiple datasets originating from independent studies using multiple asthma tissue/ cell sample types (bronchial epithelium, peripheral blood, CD4+ and CD8+ lymphocytes, nasal epithelial, induced sputum, airway fibroblast and airway macrophages). To our knowledge this is the first study to identify and compare tissue-specific asthma-relevant genes and pathways from publicly available gene expression data and to utilize them for better asthma management.

### Multi-tissue transcriptomic analysis can identify appropriate surrogate samples for invasive/ difficult to collect samples

Although it is ideal to determine gene expression from target tissues (*i.e.*, cells from lung tissue in asthma), this is challenging when considering the large number of samples required given the demands of statistical power. However, recently studies have demonstrated the value of focusing on surrogate target tissues/cells in predicting gene expression in tissues/cells that are challenging to access in large numbers (*i.e.*, lung tissue), which have the potential to significantly move the field forward. For example, Poole and colleagues used whole-transcriptome sequencing (RNA-Seq) to demonstrate that the nasal airway epithelium mirrors the bronchial airway (Marenholz *et al.* 2006). Their study confirmed that children with asthma have an altered nasal airway transcriptome compared with healthy controls, and these changes are reflected by differential expression in the bronchial airway. Noninvasively obtainable biological samples showing considerable pathway-level overlap with difficult to obtain disease-relevant tissues could be used as surrogates for assessing disease risk and progression. For example, in experimental data indicated that nasal epithelial cells are easier to obtain and may act as a surrogate for bronchial epithelium in asthma studies (Thavagnanam *et al.* 2014; McDougall *et al.* 2008). The accurate prediction of tissue-specific gene expression could thus provide useful information for biomarker development and drug targeting. Since nasal epithelial tissue and induced sputum are readily accessible from patients and controls, this study does suggest using these samples as a model system for asthma, as there is more pathway-level similarity to the upper airway and bronchial samples, respectively.

### Genes and networks interact across tissues and show connection to Canonical Pathways showing the hallmarks of asthma

A striking finding of our study is that the top significant DEGs do not significantly overlap between tissues, rather participate in complex pathways and networks crucial for asthma manifestation. Several of these genes are involved in cellular processes such as cell migration, airway remodeling and mucus production that are crucial for asthma manifestation. For examples, significant DEGs in our list are involved biomarkers of Th2 response (*POSTN*), inflammation (*NOS2, ALOX15*) and mucus production (MUC5AC) corroborated with a previous metanalysis of airway gene expression in asthma.(Liu *et al.* 2018)

Pathway-level overlap between multi-tissue gene transcription data shows that ‘leukocyte adhesion and diapedesis’ and ‘Th1/Th2 activation’ are top significantly enriched pathways in all samples – both from the blood and the airway compartments (Figure 4a and 4b). The roles of these pathways in the installation (recruitment of inflammatory cells from blood to airways) and persistence of inflammation is well known. Activation of IL-1beta, IL-7 and ERK signaling pathways were common across a wide range of tissue types maintaining inflammation. Signaling via Calcium-sensitive chloride channels (CLCA1) plays critical part in mucus production and hyperresponsiveness in the airways. Our study also identified Acute Phase Response Signaling pathway and Nitric Oxide and Reactive Oxygen Species (ROS) pathway activation in nasal, airway epithelial, bronchial biopsy samples that might contribute to hyperresponsiveness, while chronic inflammation as well as pro-fibrotic factors (TGF-beta signaling pathway and factors release by infiltrating eosinophils) lead to sub-epithelial fibrosis with changes of the extracellular matrix composition, smooth muscle growth, Goblet cell hyperplasia and mucus secretion, collectively known as airway remodeling. Taken together, pathway-level overlap distinctly reflects molecular features of Hyperresponsiveness, inflammation and remodeling – three hallmarks of asthma.

### Multi-tissue transcriptomic analysis can be useful for identifying candidate tissues for functional validation of GWAS-identified genes

Since GWAS identify loci rather than functional variants, most GWAS have provided limited insights into underlying mechanisms. (Gallagher and Chen-Plotkin 2018) Genome-wide association studies have nominated many genetic variants for common human traits, including diseases, but in many cases the underlying biological reason for a trait association is unknown.(Gallagher and Chen-Plotkin 2018; Gotoda 2015) Therefore, annotating the possible functional effects of genetic risk variants is important in understanding genomic data. Furthermore, the overlap between GWAS-identified genes with tissue-specific gene expression should be given highest priority for expression studies. It is notable that many differentially expressed genes were identified as genome-wide significant loci in previous GWAS for asthma. This is supported by the observation that several expression quantitative trait loci (eQTLs) are located around TH2-related loci (TGF-β, POSTN, VEGF), and that airway transcriptomics has revealed consolidation of certain expressed genes into T1-driven *vs.* T2-driven modules (Modena *et al.* 2017; Albert and Kruglyak 2015).

### Connectivity map identifies perturbagens that can potentially reverse asthma signature

DEGs identified from multiple tissues can be used for pharmacologic techniques, such as drug repurposing, to identify candidate drugs that can potentially reverse asthma signature at multi-tissue level. Using asthma-relevant DEGs, we identified perturbagens (genetic or chemical) that are primarily associated with immune function, cellular transport, regulation of transcription and inflammation.

Asthma is currently treated with pharmacologic agents such as glucocorticoids, long acting/ short acting beta-agonist in addition to most recently the use of biologics. However, the pharmacologic agents used to treat asthma may not significantly reverse/ modify the expression of asthma-relevant genes at the multi-tissue level. The current study utilized connectivity map approach to identify perturbagens that might potentially reverse asthma signature at multi-tissue level.

Perturbagens for 5 tissue types: The genetic perturbagen Kruppel-like factor 6 (KLF6) is negatively associated with asthma in airway epithelial cells, macrophages, distal airway fibroblasts, CD8+ peripheral blood lymphocytes and nasal epithelial cell transcriptomes (Table 3). KLF6 belongs to the family of Kruppel-like transcription factors that critically regulate cellular/tissue homeostasis. Association of KLF6 gene with lung function has been indicated from genomic data and from *in vitro* experiments which showed that blocking of KLF6 *in vitro* can decrease transforming growth factor β (TGF-β) production leading to airway remodeling and asthma (Mgbemena *et al.* 2011; Duan *et al.* 2014). In the context of asthma, TGF-β acts as a pro-fibrotic immunomodulatory cytokine produced by multiple cell types including macrophages, epithelial cells and fibroblasts leading to airway remodeling and inflammation (Halwani *et al.* 2011; Al-Alawi *et al.* 2014; Chen *et al.* 2016). Another Kruppel-like factor KLF3 (associated with bronchial, distal fibroblasts, CD8+ cells) has also been shown in tissue-resident memory lymphocytes in asthma (Vieira Braga *et al.* 2019). Therefore, their functional modifiers could modify/ reverse asthma signature at the multi-tissue level.

Perturbagens associated with up to 4 tissue types: Three genetic perturbagens (HOXB13, BCL10 and IFNB1) and one drug compound perturbagen (entinostat; associated with Macrophages, FibroDistal, CD8+, Nasal) are associated with four tissue types (Table 3). While *bcl-10* can modulate T cell proliferation, exogenous IFN‐β might confer resistance to rhinoviral infection in human mast cell rhinoviral infection model (Akoto *et al.* 2017). Both *BCL-10* and *IFNB1* are known to be associated with asthma status (Ramakrishnan *et al.* 2019; Klemm *et al.* 2006; Bhakta *et al.* 2018). Among the perturbagens associated with three different tissue types, we found four chemical drugs and 16 genetic perturbagen candidates that negatively correlated with asthma status, several of these are previously known for their involvement in asthma. Major functional groups include: the agents with anti-inflammatory function such as BMS-345541 (marketed by Merck KGaA), a cell-permeable quinoxaline which primarily binds IKK-2 and blocks NF-κB function, and dampens airway inflammation and remodeling in mice model (Zhu *et al.* 2018). BAMBI (Bone Morphogenetic Protein and Activin Membrane-bound Inhibitor) has been described as a TGF-β type I pseudoreceptor, which regulates Treg/TH 17 differentiation (Onichtchouk *et al.* 1999). BAMBI knock out mice model of asthma demonstrated significantly reduced airway hyperresponsiveness, pulmonary inflammation, broncho-alveolar lavage fluid (BALF) eosinophil count, as well as diminished IL-4, IL-5, IL-13 and IL-6. *In vitro* data suggested that this effect is mediated by a potential regulatory role for BAMBI in TGF-β driven Treg differentiation (Vock *et al.* 2017). BAMBI also regulates macrophages inducing the differentiation of Treg through the TGF-[beta] pathway (Vock *et al.* 2015). In addition to asthma, the enhanced plasma BAMBI level in COPD positively correlated with the increased plasma TGF-β1 levels and peripheral Th17/Treg ratio indicating the significance of TGF-beta/BAMBI pathway in pulmonary disease (Zhang *et al.* 2016). LASP1, a protein-coding gene that regulates cytoskeletal activity, is downregulated in sputum proteomics of asthmatics (Takahashi *et al.* 2018). The Role of lysyl oxidases in pathogenesis of pulmonary emphysema Lysyl oxidases regulate fibrillar collagen remodeling in idiopathic pulmonary fibrosis(Tjin *et al.* 2017) The copper-dependent lysyl oxidases play a role in the formation and accumulation of elastic fibers in the extracellular matrix and augments Pulmonary emphysema in COPD (Besiktepe *et al.* 2017). Increased fibrosis and remodeling potentially via periostin-mediated pathway. Most of the perturbagens identified dsRNA-induced changes in gene expression profiles of primary nasal and bronchial epithelial cells from patients with asthma, rhinitis and controls (Wagener *et al.* 2014). These genetic and chemical perturbagens could be regarded as novel drug candidates.

Finally, it is important to note that our analyses, and hence interpretations, are subject to limitations associated with the data available in the public domain. First, multi-origin transcriptome data from independent investigators that could not be adjusted for age, race, sex and asthma severity were used. We, therefore, could not adjust all confounders. However, to minimize heterogeneity, the datasets were analyzed individually to obtain up- and down-regulated genes. Furthermore, the following transcriptome data were removed: identifiable samples related to inhaled medication and tobacco use, nasal polyps (in case of nasal dataset), animal cells, cultured cell lines. Second, although considered as a state-of-the-art drug repurposing tool, the drug perturbation gene expression profiles in connectivity maps are derived from cancer cell lines, while the asthma transcriptomic data are *in vivo* data from human patients. This could contribute to noise in the connectivity results which should be subjected to further validation in addition to acquiring multi-tissue gene expression data from systemic asthma drugs including biologics (*e.g.*, Omalizumab). In spite of these limitations, the availability of multiple data sets from public resources allowed us to explore DEGs from various tissues to identify asthma-relevant genes specific and shared tissue/cell types. Our analysis showed that tissue-specific, but not tissue-combined analysis can resolve DEGs in principal component analysis. Therefore, our transcriptome analysis was performed in a tissue-specific manner without combining gene expression data from multiple tissues. This approach was helpful to identify asthma signatures from each tissue/ sample type and to use for potential drug repurposing. The results from this study highlight the importance of tissue specific analysis of gene expression data and that this tissue/cell-type based approach could be used to treat the disease at tissue specific or at systemic/multi-tissue level.

## Conclusions

**Figure 7:**
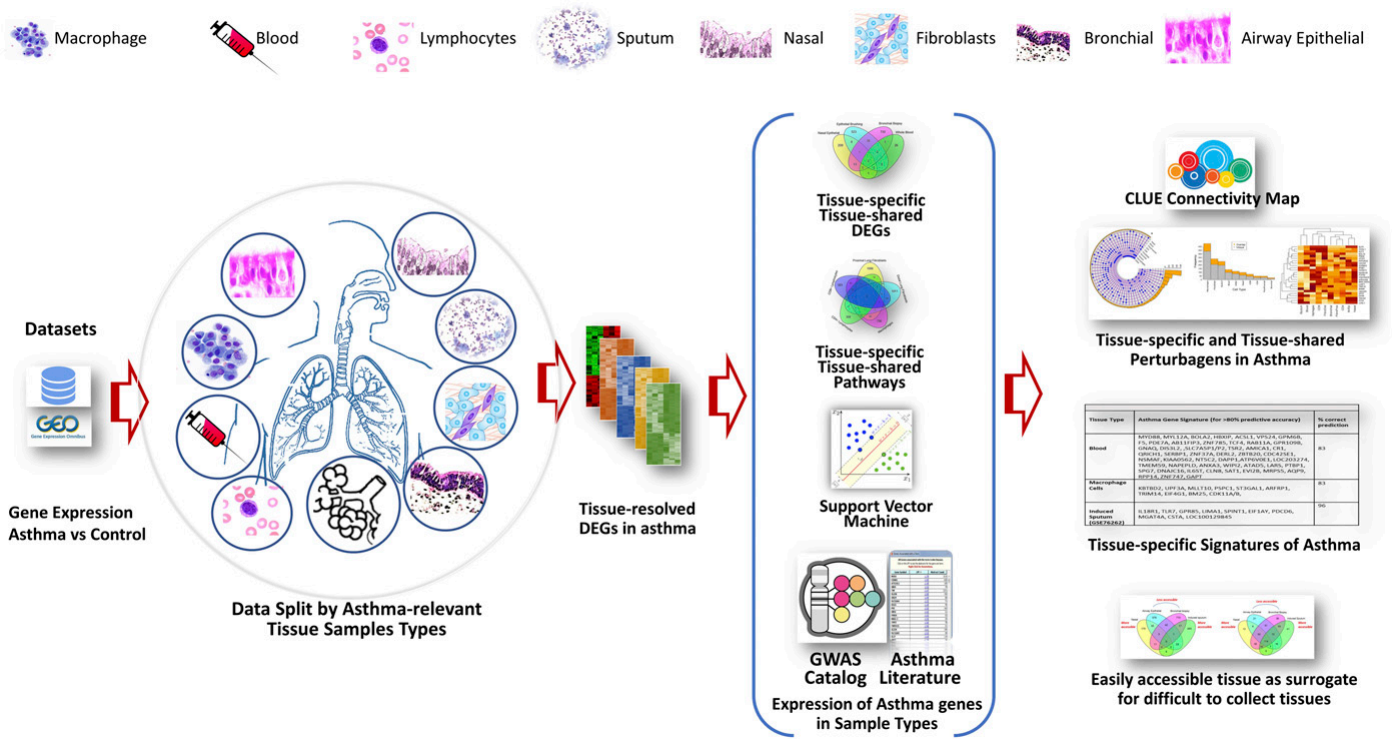
Diagramatic summary of the current study starting from the discovery of differentially regulated genes to drug repositioning in asthma using multi-tissue transcriptomic analysis. Using asthma transcriptome data identified from multiple sample types such as blood, lung, isolated fibroblasts, nasal epithelium, lymphocytes, and airway macrophages we identified molecular signatures of asthma. The signatures were further connected with GWAS-identified asthma genes. The tissue-resolved molecular signatures were further evaluated for their utility in drug repurposing (i.e. identifying perturbagens via connectivity map analysis to reverse asthma signature).

Our study utilized public domain gene expression data to capture an integrated multi-tissue snapshot of asthma-relevant genes and pathways. This is, to our knowledge, the first report utilizing multi-tissue transcriptomics for drug repurposing to manage asthma as depicted in Figure 7. The results indicated that (1) Asthma, although primarily affects the lungs, can be associated with tissue-specific DEGs and signatures as seen in other systemic diseases; (2) Major significant signature genes identified from each tissue/ sample type have previously-documented connections with asthma; (3) DEGs rarely overlap between tissues, but interact at the pathway level to manifest asthma. Top significant networks show distinct functional roles including bronchial hyperresponsiveness, inflammation and remodeling – three major hallmarks of asthma. Since asthma transcriptome signature depends on tissue (source of RNA), it is important to mention source tissue while presenting a gene signature; (4) Our study represents a model of tissue-resolved asthma transcriptome which can be used for (i) mapping asthma-relevant genes/ pathways in tissues and their functional interactions (ii) to find appropriate surrogate samples for difficult to obtain tissues (iii) to select appropriate tissue for functional studies of GWAS-identified genes (iv) to use in rational drug development such as connectivity map analysis; (5) Although major networks shows their functional relevance to asthma, limited per-sample demographic/ clinical data are currently available in the public domain. Therefore, future studies should focus on collecting transcriptomic data from multiple sample types, age and race groups, genetic background, disease subtypes and better annotated data should be made available in the public domain. Taken together, while previous studies have used different samples types to identify asthma-relevant genes and pathways, the present work has demonstrated the utility of multi-tissue gene expression data as parts of a puzzle to obtain an integrated overview of asthma.
